# Autophagy in Acute Pancreatitis: Organelle Interaction and microRNA Regulation

**DOI:** 10.1155/2021/8811935

**Published:** 2021-02-05

**Authors:** Xiaohui Yuan, Jun Wu, Xin Guo, Wei Li, Chen Luo, Shuai Li, Bing Wang, Lijun Tang, Hongyu Sun

**Affiliations:** ^1^Laboratory of Basic Medical Sciences, The General Hospital of Western Theater Command, Chengdu 610083, China; ^2^Department of General Surgery & Pancreatic Injury and Repair Key Laboratory of Sichuan Province, The General Hospital of Western Theater Command, Chengdu 610083, China; ^3^College of Medicine, Southwest Jiaotong University, Chengdu 610031, China; ^4^Department of Hepatopancreatobiliary Surgery, Panzhihua Central Hospital, Panzhihua 617000, China

## Abstract

Acute pancreatitis (AP) is a common disorder with significant hospital admission and mortality. Due to the unclarified pathological mechanism, there is still no effective and specific treatment for AP. Recently, autophagy has been found to be closely related with occurrence and development of AP, which is crucial in determining its severity and outcomes. Emerging evidence indicates that autophagy can be regulated and influenced by microRNAs and organelles, including mitochondria, endoplasmic reticulum and lysosome, through various ways in AP. Of note, the complex interplays and close relationships among autophagy, microRNA and organelles in AP are vital for figuring out pathogenesis but not clear yet. Thus, this review summarizes the role of autophagy in the pathological mechanism of AP, especially the relationship between impaired autophagy and organelles, and discusses the regulatory mechanism of microRNA on autophagy, which could offer new insights into understanding the pathogenesis of AP and developing new potential therapeutic targets against AP.

## 1. Introduction

Acute pancreatitis (AP), characterized by an inflammatory disorder of the pancreas, is the second highest cause of total hospital stays, the largest contributor to aggregate costs, and the fifth leading cause of in-hospital deaths [1], with increasing global incidence [2]. Although the majority of patients have a mild AP course with less than 1% of mortality, there is still up to 20% of AP that will be further developed to severe acute pancreatitis (SAP) accompanied by systemic inflammatory response syndrome (SIRS) and the subsequent multiple organ dysfunction and/or pancreatic necrosis, with approximately 10% of mortality [3, 4]. In addition, as high as 18% of AP patients would experience recurrence and about 8% of AP further developed into chronic pancreatitis [5, 6]. Although a great progress has been made in the understanding of AP, its pathogenesis has not been fully elucidated yet.

Autophagy is a dynamically balanced and evolutionally highly conservative process, widely existing in cells [7, 8]. Damaged and aging organelles, misfolded proteins and lipids can be degraded through autophagy, and the degradation products such as amino acids can be further reused by cells. Autophagy plays a vital role in maintaining cell homeostasis during starvation [9, 10]. According to the different ways of transporting substrates to lysosomes, there are three types of autophagy: macroautophagy, microautophagy and chaperone mediated autophagy (CMA) [11].

Macroautophagy, being most widely studied, is the main form of autophagy, including in AP. It starts from the formation and elongation of the membrane, and then turns to be the double membrane autophagosomes, which sequester organelles and long-lived proteins to be degraded. The double-membrane autophagosomes then fuse with lysosomes through the specific identification and combination between light chain3 (LC3) on autophagosome and lysosome associated membrane protein-2 (LAMP-2) on lysosome, ultimately forming the single-membraned autolysosomes. Then the cargo from autophagosome is degraded by matured hydrolases in lysosome and the degradation products are recycled back to cytoplasm. Efficient autophagy flux promotes survival of cells and plays a protective role. When normal progress of autophagy is impaired, there will be accumulation of autophagic vesicles in the cytoplasm, which damage cell homeostasis and contribute to a variety of pathological states [7, 8, 11, 12]. The autophagy mentioned in this review is all macroautophagy.

In recent years, autophagy has been confirmed to play an important role in the progression of many diseases, such as neurodegenerative diseases [13, 14], cardiomyopathy [15, 16], fatty liver [17] and cancers [18]. It is noteworthy that the autophagy flux of the exocrine pancreas is higher than liver, kidney, heart or endocrine pancreas in mammals [19], implying the crucial role of autophagy in the pathological mechanism of AP. Therefore, this review summarizes the role of autophagy in the pathological mechanism of AP, especially the relationship between impaired autophagy and organelles. In addition, due to the importance of microRNA (miRNA) in regulating human physiological and pathological processes, we discuss the regulatory mechanism of microRNA on autophagy. miRNA regulation on autophagy and the interactions between organelles and autophagy regulation are important to understand the pathogenesis of AP and offer new insights into developing new potential therapeutic targets against AP.

## 2. Autophagy and Cellular Organelles in AP

In pancreatic acinar cells of experimental animal AP models and human AP patients, the accumulation of a large number of vacuoles has been observed long before and is considered to be the characteristic of acinar cells in AP, which has been proved to be the abnormal autophagy related vacuoles. Because of the impaired process of autophagy, the long-term accumulated vacuoles can fuse with each other to form larger vacuoles than normal autophagic vacuoles [19–22]. In rodent models of AP, vacuolization is suggested to be induced by impaired autophagy which hindered the degradation of long-lived proteins, resulting in overactivation of trypsinogen of pancreatic acinar cells. All these above have proved that autophagy is involved in the pathogenesis of AP, but its mechanism has not been fully understood [23]. Under physical conditions, organelles in pancreatic acinar cells, including endoplasmic reticulum (ER), mitochondria, lysosomes, play a very important role in the production, storage and secretion process of digestive enzyme. However, during AP, organelles dysfunction occur and cause the activation of trypsinogen, and participate in the impaired autophagy flux [24]. The relationship between impaired autophagy and organelle dysfunction is complex and mutually affected [25–27]. What's more, the selective-macroautophagy can recognize and degrade specific substrates such as the disordered, damaged or aging organelles [28, 29]. Therefore, here, we discuss the specific mechanism of the interaction between autophagy and each organelle, respectively.

### 2.1. Autophagy and Mitochondria in AP

Mitochondria is a cellular organelle with double membrane structure containing circular genome, namely the mitochondrial DNA (mtDNA), whose normal physiological functions are important for the survival of cells [30]. On the function, mitochondria produce ATP mainly for cells through oxidative phosphorylation (OXPHOS), which is mediated by the electron transport chain (ETC) located on the inner mitochondrial membrane. The electrochemical gradient generated by ETC through a series of redox reactions drives the production of ATP and also generates mitochondrial membrane potential (MMP), which is very important for maintaining the physical function of mitochondria [31, 32]. Mitochondrial membrane permeability transition pore (MPTP), a non-regulatory pore, is composed of multiple proteins and connected with both the inner and outer mitochondrial membranes, and the solute with molecular weight smaller than 1500 Da and water can enter the mitochondrial matrix through the MPTP. But continuous opening of MPTP will lead to the decline of membrane potential, which in turnl lead to the decrease of ATP production and ultimate the mitochondrial dysfunction [33]. Cyclophilin D (CypD) is a key protein to control the opening of PTP. Inhibition of CypD by using drugs or genetic blocking can shut down PTP and restore mitochondrial function [34]. Mitochondrial dysfunction and corresponding morphological changes such as mitochondrial swelling, decreased cristae, etc. could be observed in acinar cells during AP [35].

Mitochondrial dysfunction can lead to the impaired process of autophagy mediated by CypD related PTP opening. In many kinds of AP animal models, mitochondrial dysfunction in pancreatic acinar cells occurs through Ca2+ dependent or Ca2 + independent pathways, but they all converge on continuous opening of CypD-related PTP [35]. The reason for PTP opening in Arginine-induced AP was the decrease of ATP synthase activity [36], and the increase of Ca2+ in AP induced by caerulein and bile acid accounted for the opening of PTP [37], while the PTP opening in alcoholic AP was mediated by the decline in Nicotinamide adenine dinucleotide (NAD) [27]. Rajarshi et al. [34] established CEA-AP by intraperitoneal injection of caerulein to C57BL/6 mice, and treated acinar cells isolated from the mouse pancreas with cholecystokinin (CCK) to induce AP at the level of cell. They found that the continuous opening of mitochondrial PTP would block the autophagy process due to the decrease of ATP, and the impaired autophagy then induced the activation of trypsin. While ATP production can be restored through the inhibition of MPTP opening, thereby increasing the efficiency of autophagy, reducing trypsin activation and alleviating AP. In 2018, Biczo et al. [35] established different AP models in a variety of animals through various methods, and found that mitochondrial dysfunction plays a central role in the pathogenesis of AP. Among these, by intraperitoneal injection of arginine (Arg) to rats and mice, respectively, to create Arg-AP model, they found that free Arg in the mitochondria of the pancreatic tissue increased sharply, and was degraded through the pathway of ornithine rather than NO and then degradation product worked on the ATP synthase, leading to mitochondrial dysfunction. Impaired autophagy, as the downstream events of mitochondrial dysfunction, could cause ER stress and lipid metabolism disorders and eventually result in AP. However, through knocking out the CypD gene, mitochondrial dysregulation was corrected, and its downstream response like impaired autophagy was improved, and AP was alleviated. Besides, the results of human AP pancreas were consistent with the experimental outcomes. Therefore, mitochondrion dysfunction causes impaired autophagy flux mainly by the role of CypD related PTP opening in experimental AP models.

Meanwhile, impaired autophagy can also influence mitochondrion by inefficient clearance of dysfunction mitochondrion. Damaged mitochondria are transported to lysosomes for degradation through selective autophagy, a process known as mitophagy, which is a protective mechanism of cells [38]. Mitophagy can be activated by mitochondrial dysfunction occurring in AP [39, 40]. However, the knockout of ATG5 and ATG7 genes could inhibit autophagy including mitophagy and lead to the accumulation of dysfunctional mitochondria [25, 41]. Mitochondrial dysfunction in AP causes inefficient autophagy, and the impaired autophagy in turn fails to degrade these dysfunctional mitochondria timely, further aggravating the mitochondrial dysfunction and the problem of ATP production. Consequently, mitochondrial dysfunction and abnormal autophagy would form a vicious cycle, and these two pathological conditions existed persistently and were involved in the pathogenesis of AP [27].

### 2.2. Autophagy and ER in AP

ER is an extensive membranous labyrinth network structure with branching tubes and flattened sacs existing in all eukaryotic cells [42]. ER can regulate the synthesis of proteins and lipids, store Ca^2+^ and regulate the concentration of Ca^2+^ in cells [24]. The protein synthesis and the number of ER in the pancreatic acinar cells are higher compared with other type of cells [43] Therefore the stable physiological function of the ER is particularly important for the homeostasis and survival of acinar cells. When the amount of protein folding exceeds the capacity of the ER due to various causes, the normal physiological state of ER will be disrupted. This status is termed as the endoplasmic reticulum stress (ER stress), which is characterized by the accumulation of unfolded and misfolded proteins and the disorder of Ca^2+^ balance. In order restore the function of ER, cells would activate unfolded protein response (UPR), and UPR is induced by activating three sensors located in ER: PERK (double-stranded RNA-dependent protein kinase (PKR)-like ER kinase), IRE1 (inositol-requiring 1*α*) and ATF6 (activating transcription factor 6) [44–47]. Morphological changes of ER can be observed at the early stage of AP [48], such as swollen ER, vacuolation, loss of ribosome, etc. [43], indicating that ER stress participates in pathological mechanism of AP.

Actually, ER is closely related to autophagy in different stages. One of the membrane sources of autophagosome is the rough endoplasmic reticulum [39, 49], and both the initiation and maturation of autophagosome have a close relationship with ER [50]. So, when the homeostasis of ER is disrupted, the effective autophagy will be interrupted or the situation of already impaired autophagy will be worsened. Xu et al. [51], using the AR42J cell line and mice with caerulein-induced AP, found that IL-1*β* can disrupt ER homeostasis, cause ER stress, and release large amounts of Ca^2+^ from ER into the cytoplasm, leading to impaired autophagy. The induction of impaired autophagy was depended on Ca^2+^ released from ER and led to the activation of trypsin. Dolai et al. [52] half knocked out the mammalian uncoordinated-18c (Munc18c) gene, which is located in the basolateral plasma membrane of pancreatic acinar cells [53] and play a role in exocrine fusion in cells [54], in C57BL/6 mice and established Munc18c-knockout human acinar cells using lentivirus, and then induced AP in mice, isolated acinar cells and Munc18c-knockout human acinar cells with caerulein and CCK. In their study, they found that after the knockout of Munc18c, the exocytosis of apical side of acinar cells stimulated by the normal physiological doses of CCK was not affected, but the exocytosis of the basolateral plasma membrane was blocked. Thus, lack of Munc 18c increased the burden of cellular degradation pathway when treated with excessive stimuli of CCK, caused ER stress, and increased autophagy induction mainly through the PERK/eukaryotic initiation factor 2*α* (eIF2*α*) pathway [55]. Under the background of already impaired autophagy process by overdose of CCK, the accumulation of autophagic vacuoles in acinar cells was further worsened by Munc18c's effect on exocytosis of the basolateral plasma membrane. And alcohol-induced ER stress can affect the folding and transportation of lysosomal proteases and lysosomal membrane proteins such as LAMP2, which would next hinder the normal process of autophagy and finally cause alcohol-induced AP [56]. Besides, Chen et al. [57] found that in AP, melatonin inhibited the inflammatory response by inhibiting ER stress, and finally promoted autophagy to play a protective role for acinar cells. The restoration of ER functions couldin turn promoted autophagy.

There is a crosstalk between UPR and autophagic pathways because both of them are aiming at restoring ER function, with macroautophagy acting as a protein degradation system for misfolded proteins and damaged ER [56]. Fazio et al. [58] upregulated the expression of stanniocalcin 2 (STC2) in C57BL/6 mice by transgenic technology to study AP induced by caerulein. It was found that STC2, a secreted glycoprotein [59], increased in expression along with the activation of PERK within 4 hours after the onset of pancreatic injury, and had close relations to UPR. STC2 overexpression resulted in the reduction of PERK activation due to negative feedback, then decreased phosphorylation of PERK and eIF2*α* in the mice pancreas, but increased the expression of ATF4 (activating transcription factor 4, a downstream molecule of eIF2*α* in PERK signaling pathway of UPR) and autophagy induction, indicating that the increased ATF4 might be the reason for elevated induction of autophagy and eventually reduce the pathological damage in AP. And ATF4 in mice acini was also found to induce the occurrence of autophagy. What's more, there were experiments proving that p-eIF2*α* and ATF4 are necessary for autophagy induction in vitro [60–62]. In addition, some proteins in adequate UPR can help to stabilize the autophagy flux. For instance, X-box binding protein1 (XBP1), a downstream molecule of IRE1 in UPR signaling pathways, could inhibit the accumulation of autophagic vacuoles through inhibiting the autophagy induction and facilitating the processing of cathepsins [63–66]. Furthermore, ATF4 and ATF6 in UPR can activate the transcription of autophagy genes, and the up-regulation of them can promote the initiation of autophagy [67, 68]. UPR and autophagy both can help restore the function of ER.

The situation of autophagy can influence and determine the condition of ER. Vacuoles accumulation caused by impaired autophagy can cause and aggravate pathological ER stress in AP [69]. Therefore, the interruption of the normal physiological process of autophagy in turn affects ER homeostasis. Li et al. [70] found that the knockout of I*κ*B kinase *α* (IKK*α*) genecaused the impairment of autophagy and thereby led to P62 accumulation, resulting in accumulation of misfolded proteins in ER, triggering ER stress and eventually causing spontaneous pancreatitis. After knocking out of P62 gene, all these damages were alleviated [71], demonstrating that autophagy damage can indeed cause ER stress. Antonucci et al. [25] found that autophagosomes in pancreatic acinar cells cannot normally be formed due to the lack of necessary ATG7 by using Pdx1-Cre mice with the knockout of ATG7 gene. Autophagy and the degradation process of autophagic proteins which were ought to be degraded through autophagy had been suppressed, as shown by the accumulation of P62, autophagic degradation substrate proteins and the unmodified form of LC3. All these could induce ER stress and UPR, and protein synthesis was reduced due to ER injury, which eventually resulted in damage and inflammation of acinar cells. And the AP injury caused by lack of ATG7 cannot be alleviated by knocking out P62 gene. This indicates that in cells, especially acinar cells with high rate of protein synthesis, the effective autophagy flux is of great importance for maintaining the stability of ER function and the continuous recycle of misfolded proteins. Biczo et al. [35] also found that during AP, ER stress was the downstream pathological phenomena of impaired autophagy. Trehalose, which can increase autophagy activity and restore autophagy flux, was intraperitoneally injected into wild type mice in two weeks before AP being induced. They found that autophagic activity was enhanced in Arg-AP treated with trehalose, and along with the improvement of autophagy, ER stress was reduced, leading togreatly alleviated AP. This proved that impaired autophagy would disturb the balance of ER and cause the ER stress. Recently, Mareninova et al. [72] found that as a method used in the study of autophagy, transgenic green fluorescent protein (GFP) -LC3 affected autophagy in exocrine pancreas. The influence of using GFP-LC3 on autophagy eventually aggravated AP. Compared with the wild type (WT) group of AP, ER morphological changes and ER stress were more apparent in acinar cells of GFP-LC3 group, confirming that abnormal autophagy could affect the status of ER. Moreover, autophagy can selectively engulf ER filled with misfolded proteins and lipids and degrade them in autolysosomes, and this process is called reticulophagy, a backup for inadequate ER associated degradation aiming at restoring ER function [28, 73].

### 2.3. Autophagy and Lysosome in AP

Lysosome composes of acidic lumen and the cholesterol-poor lipid membrane around the lumen [74]. The inner side of lysosome membrane has thick glycocalyx along the perimeter, and this structure can prevent its membrane from being degraded by lysosomal acid hydrolases. This can ensure the separation of lysosome acid environment from other parts in cell [75], which is vital to cells as the rupture of lysosome membrane will cause the leakage of acidic contents into cells, endanger cells, and even cause cell death [76]. There are about 50 kinds of hydrolases contained in lysosomes that degrade specific substrates, including proteases, lipases, nucleases, glycosidases, phospholipases, phosphatases, and sulfate enzymes, which are usually most active at low pH levels [19]. Thus, the main function of lysosome is degradation besides the functions of secretion and signaling [75]. Among these hydrolases, the cathepsin includes serine, aspartic acid, and the main cysteine cathepsin (like cathepsin B and cathepsin L, Cat B and Cat L), which are important for lysosomes, autophagy, and other functions [12].

Lysosomes directly participate in autophagy, and the status of lysosomes can determine whether the autophagy process is effective or blocked. The pathological mechanism of impaired autophagy is different depending on the various abnormal parts. Here, the three pathological mechanisms of abnormal lysosome induced impaired autophagy are described below, including the blocked fusion of autophagosome and lysosome, abnormal cathepsins in lysosomes and inadequate synthesis of lysosomes.

#### 2.3.1. The Blocked Fusion of Autophagosome and Lysosome

LAMPs are high glycosylated transmembrane proteins, accounting for 70 percent of lysosomal membrane proteins, and play important roles in maintaining lysosomal function [77, 78]. Decrease or absence of LAMP-2 on lysosomal membrane would block the fusion of lysosomes and autophagosomes, and a large number of double-membrane autophagosomes containing undegraded substances will emerge in cells. These accumulated autophagosomes affected the normal function of the cells and participated in the occurrence and development of many diseases [79]. Fortunato et al. [79] showed that alcohol and endotoxemia depleted LAMP-2 proteins in rat pancreatic tissue, leading to accumulation of autophagosomes and a shift of cell death from apoptosis to necrosis, further promoting inflammatory responses and causing acute and chronic pancreatitis. Importantly, patients with alcoholic pancreatitis also showed local LAMP-2 depletion. Thus, it can be seen that the impaired autophagy caused by the lack of LAMP-2 expression plays a vital role in the pathogenesis or aggravation of AP and other various diseases. However, the detailed mechanisms are not fully elucidated yet, so more studies are expected. And decreased biogenesis of lysosomes can also cause the decreased expression and even lack of LAMP-2, so these two conditions should be separated in future studied.

#### 2.3.2. Abnormal Cathepsins in Lysosomes

The efficiency of autophagy flux is mainly determined by the formation rate and degradation activity of autolysosomes, and the latter factor is regulated by the level of lysosomal hydrolases, protein hydrolysis activity, pH value of lysosomes and other factors. Pancreatitis has effects on the maturation process of cathepsin in lysosomes of pancreatic acini. And the activity and degradation capacity of immature form of cathepsins are greatly reduced compared with the mature ones, resulting in the accumulation of single-membraned autolysosomes containing undegraded substrates including zymogen granule [19]. Studies have demonstrated that impaired autophagy was associated with imbalance between Cat L and Cat B. Cat L is in charge of the degradation of trypsinogen and trypsin, while Cat B converts trypsinogen to trypsin and causes trypsin accumulation within the acinar cells. Pharmacological inhibition of Cat L increased the number of active trypsin in acini, and trypsinogen activation markers were part located in autophagic vacuoles [23]. These suggested that degradation defects of lysosome might be the main mechanisms underlying increased activity of trypsin in acinar cells of pancreatitis.

#### 2.3.3. Inadequate Synthesis of Lysosomes

Recently, some studies have been conducted on the role of insufficient lysosomal synthesis in impaired autophagy of AP, which is a new research aspect for the abnormal autophagy involved in the pathological mechanism of AP. Transcription factor EB (TFEB) is a major regulator of lysosome biogenesis [80]. Wang et al. [81] established AP animal models by intraperitoneally injecting C57BL/6 J mice with caerulein, and found that caerulein activated the mechanistic target of rapamycin kinase (mTOR) of pancreas and increased degradation of TFEB. Because of the reduced number of TFEB, the number of lysosomes was declined, thus resulting in the lack of autolysosomes and ultimately causing AP. TFEB is an important factor for both lysosome and autophagy, which deserves to explore more in the future.

In addition, when lysosomes ever involved in autophagy are damaged or aging, they can be specifically identified and phagocytized by the autophagosomes and then fused with the intact lysosomes. Damaged lysosomes can be degraded as substrates through this process called lysophagy, which can protect cells [82]. Interestingly, there were also studies indicated that damaged lysosomes can recover their low pH and degradation capacity through autophagy [76].

The lysosomal synthesis, maturation of contained enzymes and fusion capacity with autophagosome all contribute to the impaired autophagic flux and further induce AP. There may be more for us to explore in this complex and vital organelle, as lysosome is so close with autophagy.

## 3. The Role of miRNAs in Regulating Autophagy of AP

miRNAs are a class of common conserved endogenous non-coding RNAs composed of 22-25 nucleotides, which are widely expressed in different species and play an important role in cell proliferation, immune response and homeostasis maintenance [83–85]. miRNA can regulate protein-coding genes by targeting a sequence in the 3'-UTR region of target gene and affect the translation and expression of proteins [86–88]. A single miRNA can simultaneously regulate multiple target genes in the genetic network, producing a strong cumulative effect on the gene network, and it is believed that they jointly regulated one third of the genes in the genome. miRNAs have already been shown to be associated with many biological processes and human diseases, and been extensively studied as new targets of clinical diagnosis and therapy [89–91].

**Figure 1:**
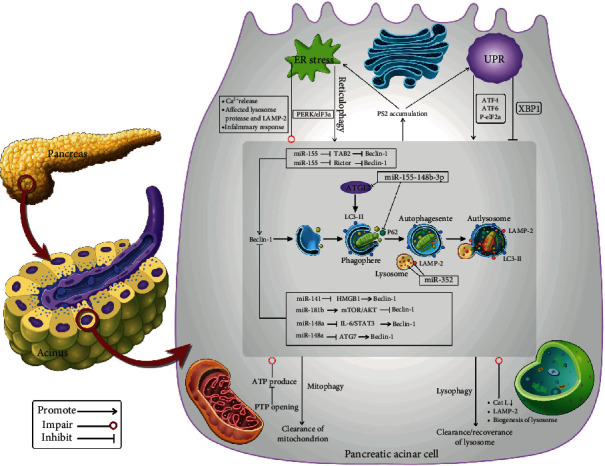
Interplays between organelles and autophagy and the regulatory mechanism of miRNAs on autophagy flux in pancreatic acinar cells. Close relations exist between each organelle and autophagy during AP. 1. Mitochondrion and autophagy: decreased ATP synthase activity, increased Ca2+, and decreased NAP all contribute to continuous PTP opening and then decreased production of ATP, leading to an impaired process of autophagy. 2. ER and autophagy: (1) IL-1*β* and alcohol-caused ER stress leads to release Ca2+ from ER and affected folding and transportation process of lysosome protease and LAMP-2, respectively, finally resulting in the hindered autophagic process. Melatonin inhibits ER stress through inhibiting the inflammatory response and then promoted autophagy. Increased burden of cellular degradation caused by lack of Munc 18c under excessive stimuli of CCK causes ER stress and then increased autophagic induction mediated by PERK/eIF2*α*; ATF4, ATF6, and p-eIF2*α* promote induction of autophagy, while XBP1 decreases the numbers of accumulated autophagic vacuoles by inhibiting autophagic induction and facilitating the processing of cathepsins. (2) Impaired autophagy by IKK*α* knockout and inhibited autophagy by ATG7 knockout both cause P62 accumulation, leading to ER stress and UPR. 3. Lysosome and autophagy. Decreased Cat L, alcohol, and endotoxemia caused depleted LAMP-2, and decline in the biogenesis of lysosome mediated by reduced TFEB all lead to impaired autophagy. 4. Aged and dysfunctional organelles (mitochondrion, ER, and lysosome) can be cleared by autophagy through mitophagy, reticulophagy, and lysophagy, respectively. miRNAs participate in the regulatory mechanisms of autophagy in AP. 1. miR155 inhibits TAB2 or Rictor, which can inhibit Beclin-1, and then promotes induction of autophagy. 2. miR-141/HMGB1, miR-181b/mTOR/AKT, miR-148a/IL-6/STAT3, and miR-375/ATG7 all contributed to the inhibition of autophagic induction mediated by inhibiting expression of Beclin-1. 3. The suppressed expression of ATG12 and P62 by miR-148b-3p and downregulated expression of LAMP-2 and amount of mature Cat L1 by miR-352 cause impaired autophagy. Based on the above, we can see strong relationships among miRNAs, organelles, and autophagy, but direct evidence is lacking about them in AP now.

Autophagy have been proved to be regulated by miRNAs at different stages: miR-376b [92], miR-17-5p [93], miR-216a [94], and miR-30b [95] can inhibit the initial formation stage of autophagosomes by inhibiting the expression of beclin1. miR-204 [96, 97] inhibits the elongation stage by working directly on LC3. miR-101 [98], miR-34a [99], miR-24-3p [100] and miR-376b [92] are able to regulate ATG4. Upregulation of miR-423-5p [101] hinders maturation of autophagosome by inhibiting autophagosome-lysosome fusion in macrophages. Recently, accumulating evidence reveals that miRNAs exert various functional roles in regulating autophagy of AP (Figure 1). Understanding the regulatory mechanism of autophagy by miRNA in AP pathogenesis is helpful for developing targeted therapies and improve clinical management of patients with AP. Here, we summarize the confirmed miRNAs that involved in the regulation of autophagic process in AP as follows.

### 3.1. miR-141

miR-141 restrained autophagy in AP during the formation process of autophagosomes through the HMGB1/Beclin-1 pathway. Zhu et al. [102] injected miR-141 loaded by adenovirus into AP mice (intraperitoneal injection of l-arginine) through the tail vein, and found that the local damage of the pancreatic tissue was significantly reduced. The outcomes of AP animal group treated by miR-141 showed the reduced autophagosomes and autolysosomes under the electron microscope compared with AP group. And molecular experiment results were consistent with the morphological observation: the expression of LC3-II (a marker protein of autophagic vesicle) was declined, and P62 (a chaperonin delivered ubiquitin substrate into the autophagic degradation pathway) was increased, but LAMP-2 did not show any statistical significance between the two groups. Subsequently, it was found that miR-141 affected protein translation by binding to the 3'UTR region of high mobility group box 1 (HMGB1) mRNA, resulting in a decrease in the expression level of downstream protein beclin-1. HMGB1 is a conserved nuclear protein that can enhance transcription and has been found to be a key regulator of autophagy [103]. Cytoplasmic HMGB1, as a Beclin-1 binding protein, induces autophagy by isolating its inhibited protein beclin-2 [104]. Beclin-1 plays an important role in the initiation of autophagy and mediates the localization of other autophagy-related proteins on the autophagosome membrane [105]. Together, these results suggested that autophagic process was impaired in AP during the formation process of autophagosomes through the HMGB1/Beclin-1 pathway by miR-141. Thus, miR-141 was expected to be a new target for AP treatment.

### 3.2. miR-148 Family

In this family, there are two members, miR-148a and miR-148b-3p, that participate in autophagic regulatory in AP to date. Miao et al. [106] investigated the role of miR-148a on autophagy of AP by treating BALB/c mice and AR42J acini cell lines with caerulein and miR-148a mimic loaded adenovirus vectors. The results revealed that the expression of miR-148 in AP was decreased. Meanwhile elevated expression of miR-148a induced by the application of miR-148a mimic could lower the increased LC3-II and Beclin1 caused by caerulein, but the expression of P62 induced by caerulein was promoted. Pancreatic pathological changes caused by caerulein were alleviated by miR-148a ultimately. These illustrated that caerulein hindered the normal process of autophagy, and miR-148a inhibited initiation of autophagy. In addition, this study also demonstrated that the inhibitory role of miR-148a on autophagy was mediated by down-regulating the interleukin-6 (IL-6)/Signal Transducers and Activators of Transcription 3 (STAT3) signaling pathway [106]. In both AP patients and AP animal models, serum IL-6 is elevated and positively correlated with the severity of disease, which can be used to predict the prognosis of disease [107]. IL-6 can activate STAT3 and autophagy enzyme gamma-aminobutyric acid (GABA) A receptor-associated protein-like 1 (GABARAPL1) in human islets [108], and play a regulatory role in the autophagy process through the mediation of STAT3 signaling pathway, exerting influence on autophagy.

Regarding miR-148b-3p, with starvation conditions autophagy was induced in AR42J cells, then Gao et al. [109] found that miR-148b-3p was significantly lowered in these treated cells. Bioinformation had predicted 593 target genes with significant differences, and multiple target genes were associated with autophagy, including ATG12 and Sqstm1. This showed that miR-148b-3p might be an important regulatory miRNA in pancreatic autophagy through direct inhibition of ATG12 and Sqstm1/p62. However, this study was mainly based on the analysis and prediction of bioinformatics, so more researches are needed to verify the role of miR-148b-3p in AP autophagy in the future.

### 3.3. miR-181b

The miR-181 family is a widely conserved miRNA group that can influence proliferation, differentiation, death and autophagy of cells [110–112]. By transfecting miR-181b mimics/inhibitors into AR42J cells, it was observed that miR-181b reduced the expression of Beclin-1 and IL3-II and inhibited autophagy. The expression level of miR-181b was significantly reduced in AP rats induced by taurocholate. While by injecting the adenovirus loaded with miR-181b through tail vein, the activation of mTOR/Akt was increased, and the expression of Beclin-1 and LC3-II and autophagy were inhibited, then the serum lipase and amylase levels of AP rats were decreased, AP was alleviated. It was further confirmed that miR-181b inhibited autophagy and reduced the damage caused by AP by activating the mTOR/Akt signaling pathway. It is by upregulating the expression of miR-181b that Panax notoginseng saponins inhibited autophagy, thus alleviating the pathology of AP and improving the survival rate of AP rats [113]. So mTOR may be the target gene of miR-181b, but more future studies are needed to verified.

### 3.4. miR-155

In 2019, Wan et al. [114] established AP model by intraperitoneally injecting BALB/C mice with caerulein. Then investigating the effect of miR-155 in AP by injecting AAV-miR-155 and AAV-miR-155 sponge into the tail vein of mice. miR-155, which was decreased in caerulein-induced AP, was revealed to inhibited its target protein MAP3K7 binding protein 2 (TAB2), while TAB2 could negatively regulated Beclin-1. And inhibited expression of miR-155 inhibited Beclin-1 through the increased expression of TAB2. Inhibited miR-155 then inhibit the induction of autophagy accompanied by reduced formation and accumulation of autophagosomes, with decreased expression of P62 and LC3II. Pathological damage of pancreas and lungs in AP mice was alleviated by the suppressed miR-155. Contrary to this result, increasing the expression of miR-155 promoted autophagy and ultimately aggravated the pathology of AP. Moreover, these results were consistent with the outcomes in SAP animal model induced by superimposed with lipopolysaccharide (LPS)/L-arginine on the basis of caerulein. Zhang et al. [115] explored the role and mechanism of miR-155 in regulating autophagy in AP at cellular level by treating AR42J cell line with caerulein. Impaired autophagy (increased expression of P62, LC3II/I and Beclin-1) and increased miR-155 were found in those treated AR42J cells. The cells were further transfected with miR-155 mimics/inhibitors and their, respectively, negative controls, and was found that miR-155 aggravated the impaired autophagy through inhibiting the expression of Rictor (RPTOR independent companion of MTOR complex 2). These studies revealed that miR-155 promote the induction of autophagy, which can be used as a potential cure target.

### 3.5. miR-375

MiR-375 is closely related to AP and is considered as a potential biomarker for SAP [116]. Recently, Zhao et al. [117] treated AR42j cell line and Wistar rats (intraperitoneal injection) with caerulein plus LPS to construct SAP models in cells and animals, and found that miR-375 expression in SAP was increased in both cells and pancreatic tissue. They took a further exploration through the transfection of miR-375 mimics/inhibitors into AR42j cell, and found that miR-375 inhibited autophagy (decreased beclin1 and LC3II/I, increased P62) through inhibiting target gene, ATG7 (autophagy related gene 7, involved in formation of autophagosomes [118]), promoted inflammatory reaction and apoptosis in acinar cells, and aggravated SAP.

### 3.6. miR-352

miR-352 is the only miRNA reported so far to regulate autophagy by acting on the lysosomal part instead of the formation process of autophagosomes like the above-mentioned miRNAs. Inducing AP in AR42J cells with taurolithocholic acid 3-sulfate (TLC-S), the expression level of miR-352 and intracellular trypsin activation increased, the expression of LAMP-2 and Cat L1 decreased, and the accumulation of intracellular vacuoles increased compared with the control group. Besides, the mRNA of LAMP-2 and Cat L1 were included in the five target genes of miR-352. In summary, miR-352 played regulatory role on autophagy in AP through two pathways. (1) With the increase of miR-352, intracellular LAMP-2 was down-regulated in expression, and the reduction or loss of LAMP-2 protein hindered the fusion between autophagosomes and lysosomes, resulting in impaired autophagy, accumulation of intracellular autophagy vesicles and an increase in trypsin activation. (2) The overexpression of miR-352 would reduce the amount of mature Cat L1, and the decrease in the active Cat L1 or damaged processing of Cat L1 reduced the clearance of trypsinogen and trypsin, thus increasing the active trypsin in cells. By inhibiting the expression of miR-352 in AP cells, the expression levels of LAMP-2 and Cat L1 were elevated, the activation of trypsin was decreased, and the cell injury was reduced. Therefore, miR-352 obstructed the autophagy process through LAMP-2 and Cat L1, increased the activation of trypsin, and was involved in the pathogenesis of AP [119].

## 4. Conclusion and Prospects

Based on the above analysis, we can confirm that the relationships between autophagy and organelles are complicated and affected mutually; and miRNAs can regulate autophagy in AP though various pathways (Figure 1). In cells, subcellular events, like autophagy, and various organelles are inseparable and interdependent functionally. There are close interplays among them, and the complex relationships among them are involved in pathological process of diseases. In this review, we summarized the recent progress about the interactions between autophagy and three organelles in AP. Autophagy can maintain physical functions of organelles and these organelles with normal functions in turn keep autophagic flux effective, and one organelle can have influences on other organelles through autophagy. However, there is still much to be explored about their role in AP and the molecular mechanisms. Understanding these interplays among them in AP will be essential for clarifying pathogenetic process of AP and development of therapeutics targeting autophagy.

miRNAs, not only predictors and biomarkers of diseases, but important regulators in plenty of biological process, are proved to participate the regulation of autophagic process in different stages, including induction of autophagic vacuoles (mainly targeted at Beclin-1), substrate targeting, lysosome fusion and degradation, even mediated by organelles. We therefore should give significant emphasis on the function of miRNA-modulated autophagy as potential therapeutic targets for AP. Actually, autophagy and organelles can be connected and exert an influence on each other via different miRNAs in various ways in many diseases such as: parkinson's disease [120], prostate cancer [121] and pancreatic ductal adenocarcinoma [122]. Therefore, when miRNAs regulate autophagy, miRNAs also affect organelles at the same time, and vice versa. So, the relationship among them in AP should be attached importance to. However, to date, the relationships among microRNA, autophagy and organelles in AP have not been studied, thus more systematic and in-depth studies are needed to explore the roles and relationships among organelles, miRNAs and autophagy in AP.

We hope the review can shed new lights on developing novel therapeutic targets and new understanding of the molecular mechanisms in AP for researchers and clinicians.

## References

[1] Lankisch P. G., Apte M., Banks P. A. (2015). Acute pancreatitis. *The Lancet*.

[2] Petrov M. S., Yadav D. (2019). Global epidemiology and holistic prevention of pancreatitis. *Nature Reviews. Gastroenterology & Hepatology*.

[3] Banks P. A., Freeman M. L., Practice Parameters Committee of the American College of Gastroenterology (2006). Practice guidelines in acute pancreatitis. *The American Journal of Gastroenterology*.

[4] van Dijk S. M., Hallensleben N. D. L., van Santvoort H. C. (2017). Acute pancreatitis: recent advances through randomised trials. *Gut*.

[5] Ahmed Ali U., Issa Y., Hagenaars J. C. (2016). Risk of recurrent pancreatitis and progression to chronic pancreatitis after a first episode of acute pancreatitis. *Clinical Gastroenterology and Hepatology*.

[6] Vipperla K., Papachristou G. I., Easler J. (2014). Risk of and factors associated with readmission after a sentinel attack of acute pancreatitis. *Clinical Gastroenterology and Hepatology*.

[7] Fortunato F., Kroemer G. (2009). Impaired autophagosome-lysosome fusion in the pathogenesis of pancreatitis. *Autophagy*.

[8] Levine B., Kroemer G. (2008). Autophagy in the pathogenesis of disease. *Cell*.

[9] Reggiori F., Komatsu M., Finley K., Simonsen A. (2012). Autophagy: more than a nonselective pathway. *International Journal of Cell Biology*.

[10] Singh R., Cuervo A. M. (2011). Autophagy in the cellular energetic balance. *Cell Metabolism*.

[11] Mizushima N. (2007). Autophagy: process and function. *Genes & Development*.

[12] Reiser J., Adair B., Reinheckel T. (2010). Specialized roles for cysteine cathepsins in health and disease. *The Journal of Clinical Investigation*.

[13] Boland B., Kumar A., Lee S. (2008). Autophagy induction and autophagosome clearance in neurons: relationship to autophagic pathology in Alzheimer's disease. *The Journal of Neuroscience*.

[14] Ma J. F., Huang Y., Chen S. D., Halliday G. (2010). Immunohistochemical evidence for macroautophagy in neurones and endothelial cells in Alzheimer's disease. *Neuropathology and Applied Neurobiology*.

[15] Nishino I., Fu J., Tanji K. (2000). Primary LAMP-2 deficiency causes X-linked vacuolar cardiomyopathy and myopathy (Danon disease). *Nature*.

[16] Bhuiyan M. S., Pattison J. S., Osinska H. (2013). Enhanced autophagy ameliorates cardiac proteinopathy. *The Journal of Clinical Investigation*.

[17] Yang L., Li P., Fu S., Calay E. S., Hotamisligil G. S. (2010). Defective hepatic autophagy in obesity promotes ER stress and causes insulin resistance. *Cell Metabolism*.

[18] Yamamoto K., Venida A., Yano J. (2020). Autophagy promotes immune evasion of pancreatic cancer by degrading MHC-I. *Nature*.

[19] Gukovskaya A. S., Gukovsky I. (2012). Autophagy and pancreatitis. *American Journal of Physiology. Gastrointestinal and Liver Physiology*.

[20] Adler G., Rohr G., Kern H. F. (1982). Alteration of membrane fusion as a cause of acute pancreatitis in the rat. *Digestive Diseases and Sciences*.

[21] Aho H. J., Nevalainen T. J., Havia V. T., Heinonen R. J., Aho A. J. (1982). Human acute pancreatitis: a light and electron microscopic study. *Acta Pathologica, Microbiologica, et Immunologica Scandinavica. Section A*.

[22] Brackett K. A., Crocket A., Joffe S. N. (1983). Ultrastructure of early development of acute pancreatitis in the rat. *Digestive Diseases and Sciences*.

[23] Mareninova O. A., Hermann K., French S. W. (2009). Impaired autophagic flux mediates acinar cell vacuole formation and trypsinogen activation in rodent models of acute pancreatitis. *The Journal of Clinical Investigation*.

[24] Gukovskaya A. S., Gorelick F. S., Groblewski G. E. (2019). Recent insights into the pathogenic mechanism of pancreatitis: role of acinar cell organelle disorders. *Pancreas*.

[25] Antonucci L., Fagman J. B., Kim J. Y. (2015). Basal autophagy maintains pancreatic acinar cell homeostasis and protein synthesis and prevents ER stress. *Proceedings of the National Academy of Sciences of the United States of America*.

[26] Habtezion A., Gukovskaya A. S., Pandol S. J. (2019). Acute pancreatitis: a multifaceted set of organelle and cellular interactions. *Gastroenterology*.

[27] Gukovsky I., Pandol S. J., Mareninova O. A., Shalbueva N., Jia W., Gukovskaya A. S. (2012). Impaired autophagy and organellar dysfunction in pancreatitis. *Journal of Gastroenterology and Hepatology*.

[28] Yao R. Q., Ren C., Xia Z. F., Yao Y. M. (2020). Organelle-specific autophagy in inflammatory diseases: a potential therapeutic target underlying the quality control of multiple organelles. *Autophagy*.

[29] Jin M., Liu X., Klionsky D. J. (2013). SnapShot: Selective autophagy. *Cell*.

[30] Duchen M. R. (2004). Mitochondria in health and disease: perspectives on a new mitochondrial biology. *Molecular Aspects of Medicine*.

[31] Nunnari J., Suomalainen A. (2012). Mitochondria: in sickness and in health. *Cell*.

[32] Sakamuru S., Attene-Ramos M. S., Xia M. (2016). Mitochondrial membrane potential assay. *Methods in Molecular Biology*.

[33] Gukovsky I., Pandol S. J., Gukovskaya A. S. (2011). Organellar dysfunction in the pathogenesis of pancreatitis. *Antioxidants & Redox Signaling*.

[34] Mukherjee R., Mareninova O. A., Odinokova I. V. (2016). Mechanism of mitochondrial permeability transition pore induction and damage in the pancreas: inhibition prevents acute pancreatitis by protecting production of ATP. *Gut*.

[35] Biczo G., Vegh E. T., Shalbueva N. (2018). Mitochondrial dysfunction, through impaired autophagy, leads to endoplasmic reticulum stress, deregulated lipid metabolism, and pancreatitis in animal models. *Gastroenterology*.

[36] Biczó G., Hegyi P., Dósa S. (2011). The crucial role of early mitochondrial injury in L-lysine-induced acute pancreatitis. *Antioxidants & Redox Signaling*.

[37] Gukovskaya A. S., Gukovsky I. (2011). Which way to die: the regulation of acinar cell death in pancreatitis by mitochondria, calcium, and reactive oxygen species. *Gastroenterology*.

[38] Youle R. J., Narendra D. P. (2011). Mechanisms of mitophagy. *Nature Reviews. Molecular Cell Biology*.

[39] Gukovskaya A. S., Gukovsky I., Algül H., Habtezion A. (2017). Autophagy, Inflammation, and Immune dysfunction in the pathogenesis of pancreatitis. *Gastroenterology*.

[40] Shirihai O. S., Song M., Dorn G. W. (2015). How mitochondrial dynamism orchestrates mitophagy. *Circulation Research*.

[41] Diakopoulos K. N., Lesina M., Wörmann S. (2015). Impaired autophagy induces chronic atrophic pancreatitis in mice via sex- and nutrition-dependent processes. *Gastroenterology*.

[42] Kaufman R. J. (1999). Stress signaling from the lumen of the endoplasmic reticulum: coordination of gene transcriptional and translational controls. *Genes & Development*.

[43] Courreges A. P., Najenson A. C., Vatta M. S., Bianciotti L. G. (2019). Atrial natriuretic peptide attenuates endoplasmic reticulum stress in experimental acute pancreatitis. *Biochimica et Biophysica Acta - Molecular Basis of Disease*.

[44] Schröder M., Kaufman R. J. (2005). The mammalian unfolded protein response. *Annual Review of Biochemistry*.

[45] Gonzalez-Teuber V., Albert-Gasco H., Auyeung V. C., Papa F. R., Mallucci G. R., Hetz C. (2019). Small molecules to improve ER Proteostasis in disease. *Trends in Pharmacological Sciences*.

[46] Zhang K., Kaufman R. J. (2008). From endoplasmic-reticulum stress to the inflammatory response. *Nature*.

[47] Hotamisligil G. S. (2010). Endoplasmic reticulum stress and the inflammatory basis of metabolic disease. *Cell*.

[48] Logsdon C. D., Ji B. (2013). The role of protein synthesis and digestive enzymes in acinar cell injury. *Nature Reviews. Gastroenterology & Hepatology*.

[49] Park S., Zuber C., Roth J. (2020). Selective autophagy of cytosolic protein aggregates involves ribosome-free rough endoplasmic reticulum. *Histochemistry and Cell Biology*.

[50] Calvo-Garrido J., Escalante R. (2014). Autophagy dysfunction and ubiquitin-positive protein aggregates in Dictyostelium cells lacking Vmp1. *Autophagy*.

[51] Xu B., Bai B., Sha S. (2014). Interleukin-1*β* induces autophagy by affecting calcium homeostasis and trypsinogen activation in pancreatic acinar cells. *International Journal of Clinical and Experimental Pathology*.

[52] Dolai S., Liang T., Orabi A. I. (2018). Munc18c's role in exocytosis, autophagy, and pancreatitis. *The Journal of Biological Chemistry*.

[53] Gaisano H. Y., Lutz M. P., Leser J. (2001). Supramaximal cholecystokinin displaces Munc18c from the pancreatic acinar basal surface, redirecting apical exocytosis to the basal membrane. *The Journal of Clinical Investigation*.

[54] Archbold J. K., Whitten A. E., Hu S. H., Collins B. M., Martin J. L. (2014). SNARE-ing the structures of Sec1/Munc18 proteins. *Current Opinion in Structural Biology*.

[55] Walter P., Ron D. (2011). The unfolded protein response: from stress pathway to homeostatic regulation. *Science*.

[56] Lugea A., Waldron R. T., French S. W., Pandol S. J. (2014). Drinking and driving pancreatitis: links between endoplasmic reticulum stress and autophagy. *Autophagy*.

[57] Chen Y., Zhang J., Zhao Q. (2016). Melatonin induces anti-inflammatory effects to play a protective role via endoplasmic reticulum stress in acute pancreatitis. *Cellular Physiology and Biochemistry*.

[58] Fazio E. N., Dimattia G. E., Chadi S. A., Kernohan K. D., Pin C. L. (2011). Stanniocalcin 2 alters PERK signalling and reduces cellular injury during cerulein induced pancreatitis in mice. *BMC Cell Biology*.

[59] Moore E. E., Kuestner R. E., Conklin D. C. (1999). Stanniocalcin 2: characterization of the protein and its localization to human pancreatic alpha cells. *Hormone and Metabolic Research*.

[60] Rzymski T., Milani M., Pike L. (2010). Regulation of autophagy by ATF4 in response to severe hypoxia. *Oncogene*.

[61] Kouroku Y., Fujita E., Tanida I. (2007). ER stress (PERK/eIF2 *α* phosphorylation) mediates the polyglutamine-induced LC3 conversion, an essential step for autophagy formation. *Cell Death and Differentiation*.

[62] Milani M., Rzymski T., Mellor H. R. (2009). The role of ATF4 stabilization and autophagy in resistance of breast cancer cells treated with Bortezomib. *Cancer Research*.

[63] Hetz C., Thielen P., Matus S. (2009). XBP-1 deficiency in the nervous system protects against amyotrophic lateral sclerosis by increasing autophagy. *Genes & Development*.

[64] Vidal R. L., Figueroa A., Court F. A. (2012). Targeting the UPR transcription factor XBP1 protects against Huntington's disease through the regulation of FoxO1 and autophagy. *Human Molecular Genetics*.

[65] Yan D., Wang H. W., Bowman R. L., Joyce J. A. (2016). STAT3 and STAT6 Signaling pathways synergize to promote Cathepsin secretion from macrophages via IRE1*α* activation. *Cell Reports*.

[66] Zhou Y., Lee J., Reno C. M. (2011). Regulation of glucose homeostasis through a XBP-1-FoxO1 interaction. *Nature Medicine*.

[67] B’chir W., Maurin A. C., Carraro V. (2013). The eIF2*α*/ATF4 pathway is essential for stress-induced autophagy gene expression. *Nucleic Acids Research*.

[68] Zalckvar E., Berissi H., Mizrachy L. (2009). DAP-kinase-mediated phosphorylation on the BH3 domain of beclin 1 promotes dissociation of beclin 1 from Bcl-XL and induction of autophagy. *EMBO Reports*.

[69] Barrera K., Stanek A., Okochi K. (2018). Acinar cell injury induced by inadequate unfolded protein response in acute pancreatitis. *World Journal of Gastrointestinal Pathophysiology*.

[70] Li N., Wu X., Holzer R. G. (2013). Loss of acinar cell IKK*α* triggers spontaneous pancreatitis in mice. *The Journal of Clinical Investigation*.

[71] Hall J. C., Crawford H. C. (2014). The conspiracy of autophagy, stress and inflammation in acute pancreatitis. *Current Opinion in Gastroenterology*.

[72] Mareninova O. A., Jia W., Gretler S. R. (2020). Transgenic expression of GFP-LC3 perturbs autophagy in exocrine pancreas and acute pancreatitis responses in mice. *Autophagy*.

[73] Bernales S., Schuck S., Walter P. (2014). ER-phagy: selective autophagy of the endoplasmic reticulum. *Autophagy*.

[74] Schulze H., Kolter T., Sandhoff K. (2009). Principles of lysosomal membrane degradation: cellular topology and biochemistry of lysosomal lipid degradation. *Biochimica et Biophysica Acta*.

[75] Settembre C., Fraldi A., Medina D. L., Ballabio A. (2013). Signals from the lysosome: a control Centre for cellular clearance and energy metabolism. *Nature Reviews. Molecular Cell Biology*.

[76] Maejima I., Takahashi A., Omori H. (2013). Autophagy sequesters damaged lysosomes to control lysosomal biogenesis and kidney injury. *The EMBO Journal*.

[77] Eskelinen E. L., Tanaka Y., Saftig P. (2003). At the acidic edge: emerging functions for lysosomal membrane proteins. *Trends in Cell Biology*.

[78] Saftig P., Klumperman J. (2009). Lysosome biogenesis and lysosomal membrane proteins: trafficking meets function. *Nature Reviews. Molecular Cell Biology*.

[79] Fortunato F., Bürgers H., Bergmann F. (2009). Impaired autolysosome formation correlates with Lamp-2 depletion: role of apoptosis, autophagy, and necrosis in pancreatitis. *Gastroenterology*.

[80] Settembre C., di Malta C., Polito V. A. (2011). TFEB links autophagy to lysosomal biogenesis. *Science*.

[81] Wang S., Ni H. M., Chao X. (2019). Impaired TFEB-mediated lysosomal biogenesis promotes the development of pancreatitis in mice and is associated with human pancreatitis. *Autophagy*.

[82] Mizushima N. (2019). The ubiquitin E2 enzyme UBE2QL1 mediates lysophagy. *EMBO Reports*.

[83] Upadhyay S., Mittal E., Philips J. A. (2018). Tuberculosis and the art of macrophage manipulation. *Pathogens and Disease*.

[84] D’Adamo S., Cetrullo S., Minguzzi M., Silvestri Y., Borzì R. M., Flamigni F. (2017). MicroRNAs and autophagy: fine players in the control of chondrocyte homeostatic activities in osteoarthritis. *Oxidative Medicine and Cellular Longevity*.

[85] Wu D., Wang H., Teng T., Duan S., Ji A., Li Y. (2018). Hydrogen sulfide and autophagy: a double edged sword. *Pharmacological Research*.

[86] Wang X., Chen H., Bai J., He A. (2017). MicroRNA: an important regulator in acute myeloid leukemia. *Cell Biology International*.

[87] Liang M., Habib Z., Sakamoto K., Chen X., Cao G. (2017). Mycobacteria and autophagy: many questions and few answers. *Current Issues in Molecular Biology*.

[88] Gozuacik D., Akkoc Y., Ozturk D. G., Kocak M. (2017). Autophagy-regulating microRNAs and Cancer. *Frontiers in Oncology*.

[89] Krol J., Loedige I., Filipowicz W. (2010). The widespread regulation of microRNA biogenesis, Function and decay. *Nature Reviews Genetics*.

[90] Hammond S. M. (2015). An overview of microRNAs. *Advanced Drug Delivery Reviews*.

[91] Zhao Y., Wang Z., Zhang W., Zhang L. (2019). MicroRNAs play an essential role in autophagy regulation in various disease phenotypes. *BioFactors*.

[92] Korkmaz G., le Sage C., Tekirdag K. A., Agami R., Gozuacik D. (2012). miR-376b controls starvation and mTOR inhibition-related autophagy by targeting ATG4C and BECN1. *Autophagy*.

[93] Chatterjee A., Chattopadhyay D., Chakrabarti G. (2014). miR-17-5p downregulation contributes to paclitaxel resistance of lung cancer cells through altering beclin1 expression. *PLoS One*.

[94] Menghini R., Casagrande V., Marino A. (2014). MiR-216a: a link between endothelial dysfunction and autophagy. *Cell Death & Disease*.

[95] Tang B., Li N., Gu J. (2014). Compromised autophagy by MIR30B benefits the intracellular survival of helicobacter pylori. *Autophagy*.

[96] Hall D. P., Cost N. G., Hegde S. (2014). TRPM3 and miR-204 establish a regulatory circuit that controls oncogenic autophagy in clear cell renal cell carcinoma. *Cancer Cell*.

[97] Mikhaylova O., Stratton Y., Hall D. (2012). VHL-regulated MiR-204 suppresses tumor growth through inhibition of LC3B-mediated autophagy in renal clear cell carcinoma. *Cancer Cell*.

[98] Frankel L. B., Wen J., Lees M. (2011). microRNA-101 is a potent inhibitor of autophagy. *The EMBO Journal*.

[99] Liu X. J., Hong Q., Wang Z., Yu Y. Y., Zou X., Xu L. H. (2015). MicroRNA-34a suppresses autophagy in tubular epithelial cells in acute kidney injury. *American Journal of Nephrology*.

[100] Pan B., Chen Y., Song H., Xu Y., Wang R., Chen L. (2015). Mir-24-3p downregulation contributes to VP16-DDP resistance in small-cell lung cancer by targetingATG4A. *Oncotarget*.

[101] Tu H., Yang S., Jiang T. (2019). Elevated pulmonary tuberculosis biomarker miR-423-5p plays critical role in the occurrence of active TB by inhibiting autophagosome-lysosome fusion. *Emerging Microbes & Infections*.

[102] Zhu H., Huang L., Zhu S. (2016). Regulation of autophagy by systemic admission of microRNA-141 to target HMGB1 in l-arginine-induced acute pancreatitis in vivo. *Pancreatology*.

[103] Tang D., Kang R., Coyne C. B., Zeh H. J., Lotze M. T. (2012). PAMPs and DAMPs: signal 0s that spur autophagy and immunity. *Immunological Reviews*.

[104] Tang D., Kang R., Livesey K. M. (2010). Endogenous HMGB1 regulates autophagy. *The Journal of Cell Biology*.

[105] New M., Van Acker T., Long J. S., Sakamaki J.-i., Ryan K. M., Tooze S. A. (2017). molecular pathways controlling autophagy in pancreatic Cancer. *Frontiers in Oncology*.

[106] Miao B., Qi W. J., Zhang S. W. (2019). miR-148a suppresses autophagy by down-regulation of IL-6/STAT3 signaling in cerulein-induced acute pancreatitis. *Pancreatology*.

[107] Staubli S. M., Oertli D., Nebiker C. A. (2015). Laboratory markers predicting severity of acute pancreatitis. *Critical Reviews in Clinical Laboratory Sciences*.

[108] Linnemann A. K., Blumer J., Marasco M. R. (2017). Interleukin 6 protects pancreatic *β* cells from apoptosis by stimulation of autophagy. *The FASEB Journal*.

[109] GAO B., WANG D., SUN W., MENG X., ZHANG W., XUE D. (2016). Differentially expressed microRNA identification and target gene function analysis in starvation-induced autophagy of AR42J pancreatic acinar cells. *Molecular Medicine Reports*.

[110] Cuesta R., Martinez-Sanchez A., Gebauer F. (2009). miR-181a regulates cap-dependent translation of p27(kip1) mRNA in myeloid cells. *Molecular and Cellular Biology*.

[111] Henao-Mejia J., Williams A., Goff L. A. (2013). The microRNA miR-181 is a critical cellular metabolic rheostat essential for NKT cell ontogenesis and lymphocyte development and homeostasis. *Immunity*.

[112] Yang Z. W., Meng X. X., Xu P. (2015). Central role of neutrophil in the pathogenesis of severe acute pancreatitis. *Journal of Cellular and Molecular Medicine*.

[113] Liu M. W., Wei R., Su M. X., Li H., Fang T. W., Zhang W. (2018). Effects of Panax notoginseng saponins on severe acute pancreatitis through the regulation of mTOR/Akt and caspase-3 signaling pathway by upregulating miR-181b expression in rats. *BMC Complementary and Alternative Medicine*.

[114] Wan J., Yang X., Ren Y. (2019). Inhibition of miR-155 reduces impaired autophagy and improves prognosis in an experimental pancreatitis mouse model. *Cell Death & Disease*.

[115] Zhang X., Chu J., Sun H. (2020). MiR-155 aggravates impaired autophagy of pancreatic acinar cells through targeting Rictor. *Acta Biochimica et Biophysica Sinica*.

[116] Calvano J., Edwards G., Hixson C., Burr H., Mangipudy R., Tirmenstein M. (2016). Serum microRNAs-217 and -375 as biomarkers of acute pancreatic injury in rats. *Toxicology*.

[117] Zhao S. P., Yu C., Xiang K. M., Yang M. S., Liu Z. L., Yang B. C. (2020). miR-375 inhibits autophagy and further promotes inflammation and apoptosis of acinar cells by targeting ATG7. *Pancreas*.

[118] Le Guo J. Z., Qu Y., Yin R. (2016). microRNA-20a inhibits Autophagic process by targeting ATG7 and ATG16L1 and Favors mycobacterial survival in macrophage cells. *Frontiers in Cellular and Infection Microbiology*.

[119] Song Z., Huang Y., Liu C. (2018). miR-352 participates in the regulation of trypsinogen activation in pancreatic acinar cells by influencing the function of autophagic lysosomes. *Oncotarget*.

[120] Exner N., Lutz A. K., Haass C., Winklhofer K. F. (2012). Mitochondrial dysfunction in Parkinson's disease: molecular mechanisms and pathophysiological consequences. *The EMBO Journal*.

[121] Sohn E. J. (2018). MicroRNA 200c-3p regulates autophagy via upregulation of endoplasmic reticulum stress in PC-3 cells. *Cancer cell International*.

[122] Kwon J. J., Willy J. A., Quirin K. A. (2016). Novel role of miR-29a in pancreatic cancer autophagy and its therapeutic potential. *Oncotarget*.

